# Association of Vision-related Quality of Life with Visual Function in Age-Related Macular Degeneration

**DOI:** 10.1038/s41598-019-51769-7

**Published:** 2019-10-25

**Authors:** Susanne G. Pondorfer, Jan. H. Terheyden, Manuel Heinemann, Maximilian W. M. Wintergerst, Frank G. Holz, Robert P. Finger

**Affiliations:** Department. of Ophthalmology, University of Bonn Ernst-Abbe-Str. 2, D-53127 Bonn, Germany

**Keywords:** Epidemiology, Retinal diseases

## Abstract

The purpose of this study was to assess which visual function measures are most strongly associated with vision-related quality of life (VRQoL) in age-related macular degeneration (AMD). A cross-sectional study of subjects with early AMD (n = 10), intermediate AMD (n = 42) and late AMD (n = 38) was conducted. Subjects were interviewed with the Impact of Vision Impairment (IVI) questionnaire. Functional tests performed included best-corrected visual acuity (BCVA), low luminance visual acuity (LLVA), visual acuity measured with the Moorfields Acuity Charts (MAC), contrast sensitivity, reading speed, mesopic and dark-adapted microperimetry. The relationship between VRQoL and visual function was assessed with multiple regressions controlling for confounders. Rasch analysis demonstrated the validity of the IVI to assess VRQoL through three subscales: reading and accessing information, mobility and independence, and emotional well-being. Subjects with late AMD had significant lower IVI scores on all subscales compared with intermediate and early AMD (p < 0.011). In the overall cohort, IVI subscales were associated with BCVA, LLVA, MAC-VA and contrast sensitivity (all p < 0.001). Among the subgroup of early and intermediate AMD subjects, reading and mobility subscales were significantly associated with MAC-VA (p < 0.013). These results suggest that MAC-VA is a useful, patient-relevant measure of visual impairment in AMD.

## Introduction

Age-related macular degeneration (AMD) is the leading cause of visual impairment in the elderly in industrial countries and an important public health problem^1,2^. Approximately 30–50 million people are affected by AMD worldwide^3^. Late stages can severely reduce visual acuity while patients with early and intermediate AMD often perform well in conventional visual function tests under high luminance and high contrast. Nevertheless, persons with early stages of AMD often complain about vision loss under low lighting, low contrast and changing light conditions, which also impacts vision-related quality of life (VRQoL)^4–6^. Standardized visual function tests under low luminance and low contrast have been met with increasing interest in particular in early stages of AMD as these tests might be more sensitive to the specific functional impairment in early and intermediate AMD^7^ than the currently most widely used outcome measure in ophthalmic research, namely high-contrast, high-luminance best-corrected visual acuity (BCVA)^8^.

However, to date we do not know which visual tests or combination of tests with or without structural data might best allow for this. From a regulatory perspective, an important pre-requisite of any functional test is its patient-relevance which can be approximated by VRQoL^9^. A validated and commonly used VRQoL instrument is the Impact of Vision Impairment (IVI) questionnaire which is reliable^10^ and has been validated psychometrically for different ocular conditions and different levels of visual acuity^9,11,12^. Therefore, in this study, we used the IVI to investigate the relationship between VRQoL and several visual functional tests under low luminance and low contrast in patients with different stages of AMD. The aim of our study was to identify which functional tests are able to discriminate between different stages of AMD and to investigate whether these tests are correlated with VRQoL in order to assess patient-relevance of the tests.

## Methods

We conducted a cross-sectional study at the Department of Ophthalmology, University of Bonn, Germany, from January 2017 until January 2019. The study was approved by the Institutional Review Board of the University Bonn (approval ID: 013/16), where patients were recruited from outpatients clinics. Written informed consent was obtained from all participants following an explanation of all tests involved. The protocol followed the tenets of the Declaration of Helsinki.

Participants were categorized into “early AMD”, “intermediate AMD” and “late AMD” based on the classification system introduced by Ferris *et al*.^13^. One eye of each patient (the more advanced eye) was included. If both eyes fitted the inclusion criteria and had the same visual acuity, the right eye was chosen. Inclusion criterion for all groups was the ability to converse, read and write German. Exclusion criteria were age <50 years, any corneal pathology that could compromise vision, amblyopia, diabetes, glaucoma, neurological or systemic disease affecting vision, refractive errors >6.00 dioptres (D) of spherical equivalent and >2.00 dioptres (D) of astigmatism. In addition to the functional tests spectral domain optical coherence tomography raster scanning was performed using a 25° × 25° scan field (49 B-scans, automated real-time mode 20 frames, centred on the fovea) as well as fundus autofluorescence and infrared reflectance imaging (Spectralis OCT2, Heidelberg Engineering, Heidelberg, Germany). All patients also underwent a clinical examination including dilated funduscopy.

### The IVI questionnaire

The IVI is an instrument to assess different dimensions of VRQoL. It consists of 28 items with three to four responses options using Likert scales, ranging from “not at all” to “a lot”. The IVI has three specific subscales: “Reading and Accessing Information” (9 items; abbreviated as reading subscale), “Mobility and Independence” (11 items; abbreviated as mobility subscale) and “Emotional Well-being” (8 items; abbreviated as emotional subscale). We used the validated German language version of the IVI^9^.

### Functional testing

All participants underwent the following visual function tests: Best-corrected visual acuity (BCVA) in Early Treatment Diabetic Retinopathy Study (ETDRS) letters, low luminance VA (LLVA), BCVA in Moorfields Acuity Chart (MAC) letters and contrast sensitivity measurement using Pelli-Robson Charts.

Visual acuity and functional tests were performed before fundus imaging. BCVA (in letters) was assessed according to the ETDRS method^14^ at a testing distance of 4 m. If the patient was unable to read the first four rows of the chart, the distance was reduced to one meter^14^. LLVA was assessed in the same manner, but with a 2.0-log unit neutral density filter that reduces luminance by 100 fold^15^ placed in the trial frame. VA measurement with a MAC chart followed the same procedure as BCVA. The MAC charts are based on the ETDRS charts and employ a high-contrast, high-pass letter design with a gray background of the same mean luminance as the letters to simulate lower contrast situations^16^. The letters are also called “vanishing optotypes”, because, for normal vision, the detection and recognition thresholds are very similar and the letters seem to disappear soon after the recognition limit has been reached^16^. Contrast sensitivity was measured using a Pelli-Robson Chart presented at 1 m distance^17–19^. To avoid fatigue patients were allowed to take small breaks (maximum five minutes) between the tests if required.

In patients with early and intermediate AMD, we additionally assessed reading speed using the International Reading Speed Texts (IReST)^20^ and macular sensitivity via mesopic and dark-adapted microperimetry. For the IReST, patients wore their best near correction and were asked to read one paragraph aloud while they were timed with a stopwatch. Mesopic and dark-adapted microperimetry were performed after pupillary dilation with 1.0% tropicamide. Macular sensitivity was measured using the modified S-MAIA device (S-MAIA, CenterVue, Padova, Italy), which performs fundus tracking using a line-scanning laser ophthalmoscope (SLO) with a super-luminescent diode illumination with a central wave light of 850 nm for mesopic testing and with an additional LED projecting red (627 nm) stimuli for dark-adapted testing. As previously described, a customized stimulus grid was used that consisted of 33 points located at 0°, 1°, 3°, 5° and 7° from fixation^21^. First, mesopic testing was performed, followed by dark-adapted testing after 30 minutes of dark adaptation while waiting in the examination room (light was switched off, light level <0.1 lx). The microperimetric outcome measure was the mean sensitivity (MS) in dB. Due to feasibility issues (fixation stability, grid centration and age/patient fatigue) patients with late AMD did not undergo microperimetry examination. We did also not test reading speed in late AMD patients as most of them did not reach the minimum required visual acuity of 55 letters^22^. All tests were performed in one eye with the non-study eye covered with an eye-patch.

### Psychometric evaluation of the IVI

Rasch analysis was used to evaluate the instrument in our cohort. It is a psychometric method that transforms ordinal scales into interval-level scales (expressed in logits)^23,24^. Item difficulty (item measure) in relation to person ability (person measure) is calculated by placing both in the same linear continuum. The ability of the scale to discriminate different strata of person ability was assessed using person separation index (PSI) and person reliability coefficient (PR)^25^. Values of >2.0 and >0.8, respectively, were considered adequate and represented the capacity of the scale to distinguish three levels of person ability^26,27^.

Unidimensionality – the ability that a scale measures a single underlying latent trait and that the items “fit” the underlying trait – was assessed in two ways. First, we determined item fit through an “infit” mean square standardized residuals statistic^28^. Values between 0.7 and 1.3 are considered acceptable, while lower or higher values may indicate redundancy or an unacceptable level of “noise” in the responses^28^. Second, the principle component analysis (PCA) of the residuals was examined to test for local independence. The PCA of residuals for the first factor should explain at least 50% of the variance and the first contrast of residuals should be <2.0 eigenvalue^29^.

The targeting of the instrument, i.e. how well item difficulty targets person ability, was assessed by visual inspection of the person-item map and the difference between person and item mean logits. A difference of >1.0 logits indicates notable mistargeting^30^.

Furthermore, each item was assessed for differential item functioning (DIF), which is a statistical method for detecting whether sample subgroups (sex and age groups) systematically respond differently to certain items, despite having similar underlying ability. A DIF contrast of >1.0 logits is notable and suggests that the item may be biased for some participants subgroups.

Person measures (in logits) were recalibrated to a 0 to 100 scale. Higher values indicate lower visual ability and indicate poorer VRQoL. Rasch analysis was performed using commercial software (Winsteps software, ver. 3.92.1.2, Chicago, IL)^31^. The Andrich rating scale model was used for analysis^29^.

### Statistical analysis

Descriptive statistics were performed to assess baseline demographic variables for the AMD groups. The nonparametric Wilcoxon rank sum test was used to compare person measures on the three subscales of the IVI in early, intermediate AMD and late AMD subjects. Univariate linear regression was carried out to assess the relationship between the person measures and demographic variables. In the overall cohort, separate univariate linear regression models against the IVI subscale person measures were performed with each of the visual function test. If the relationship between a function test and average person measures reached a p-value < 0.05 in univariate analysis, multiple regression was used to ensure that the findings were not confounded by AMD stage and age. We additionally performed a subgroup analysis, which included only patients with early and intermediate AMD. Statistical analyses were performed using the statistical software STATA^32^.

## Results

### Sociodemographic and clinical characteristics of the participants

A total of 90 participants were recruited comprising 10 patients with early AMD (11%), 42 (47%) with intermediate AMD and 38 (42%) patients with late AMD. Participants’ mean age was 73.9 ± 8.4 years and there were more female (69%) than male participants. Patients in the late AMD group were significantly older compared to early and intermediate AMD patients (p < 0.05), while patients with early and intermediate AMD were in the same age range (p = 0.96) (Table 1). All functional tests were significantly decreased in intermediate and late AMD compared to the early AMD group (all p-values < 0.001). There was no significant difference in BCVA between early and intermediate AMD (p = 0.553), as well as in reading speed (p = 0.617) and mesopic and dark-adapted microperimetry (p = 0.274 and p = 0.141). However, LLVA, MAC-VA and contrast sensitivity were significantly decreased in intermediate AMD compared to the early AMD group (p = 0.023, p = 0.041 and p < 0.001 respectively). Detailed results can be found in Table 6 in Supplement 1.

**Table 1:** Characteristics of Participants in each AMD Category.

Characteristics	AMD group		
	Early	Intermediate	Late
Mean Age (SD)	69.8 (±6.1)	69.7 (±7.7)	79.8 (±5.9)
Patients, n (eyes)	10	42	38
Women	7 (70.0%)	31 (73.8%)	62 (68.9%)

*SD = standard deviation.

### Psychometric validation of the IVI questionnaire

The data for the IVI were fitted to the Rasch model and key indicators of fit were explored. Overall, the psychometric testing supported the use of the three IVI subscales in this sample and demonstrated satisfactory PSI and PR for all subscales. No items had to be removed due to misfit or DIF. Detailed results of the Rasch analysis can be found in Supplement 2.

### Relationship between visual function tests and VRQoL

**Figure 1:**
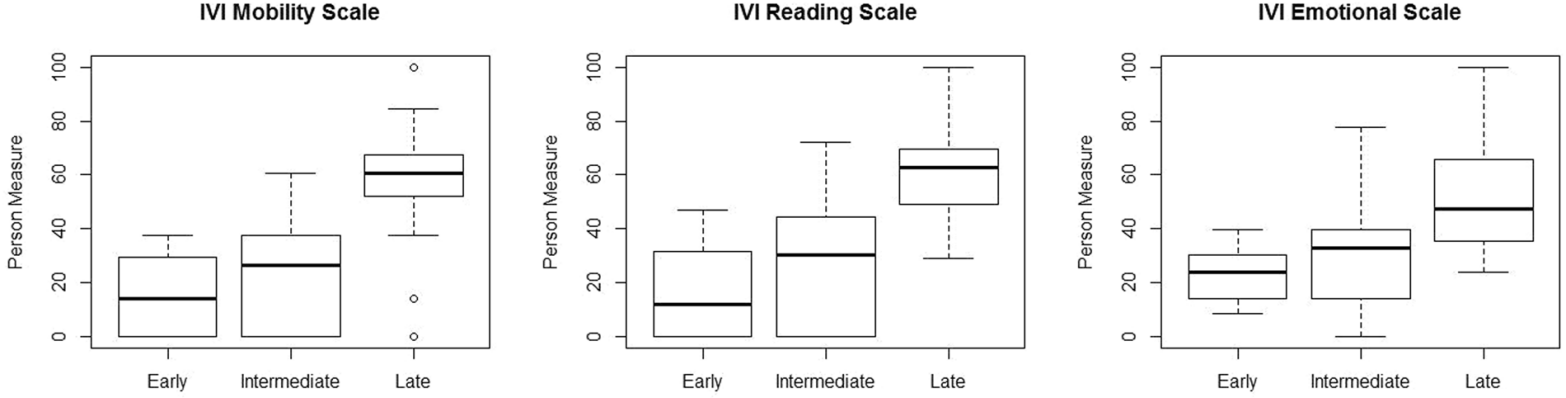
Boxplots showing IVI Reading Scale Scores, Mobility Scale Scores and Emotional Scale Scores for early, intermediate and late AMD. Each boxplot includes the maximum (upper whisker), upper quartile (top of the box), median (horizontal line in box), lower quartile (bottom of the box) and minimum (lower whisker) values.

Mean person measures (in logits) for the three subscale scores are shown in Fig. 1. Subjects with early and intermediate AMD had significantly lower person measures on all three subscales compared to subject with late AMD. There was no significant difference between early and intermediate AMD. Similarly, participants ≤75 years reported a better VRQoL compared to the older age group >75 years (Table 2). Age and AMD stage were also significantly associated with all three scales of the IVI in univariate linear regression analysis (Table 3).

**Table 2:** Baseline IVI subscales scores and comparisons between age groups, sex and AMD stage.

Variable	*n* (%)	Reading IVI		Mobility IVI		Emotional IVI	
		Mean ± SD	*P* Value	Mean ± SD	*P* Value	Mean ± SD	*P* Value
Total sample, *n* = 90		40.00 ± 25.54		36.79 ± 26.01		38.69 ± 21.62	
**Age, y**							
≤75	44 (48.8)	28.92 ± 22.39	<**0.001**	25.01 ± 21.82	<**0.001**	32.82 ± 18.19	**0.011**
>75	46 (51.1)	50.67 ± 23.96		48.06 ± 25.01		44.29 ± 23.28	
**Sex**							
Female	62 (68.9)	38.93 ± 27.02	0.662	37.46 ± 26.95	0.786	38.69 ± 22.66	0.937
Male	28 (31.1)	42.49 ± 22.18		35.29 ± 24.49		38.66 ± 19.50	
**AMD stage**							
Early	10 (11.1)	17.61 ± 19.39	<0.001*	15.27 ± 15.10	<0.001*	23.78 ± 9.71	<0.001*
Intermediate	42 (46.7)	26.97 ± 20.81		22.98 ± 19.95		30.65 ± 20.27	
Late	38 (42.2)	60.38 ± 15.58		57.71 ± 18.70		51.49 ± 18.55	

*Significant differences found between early and late AMD, and between intermediate and late AMD, but not between early and intermediate AMD; p-values based on the Wilcoxon rank sum test.

**Table 3:** Univariate Linear Regression of Baseline Demographics against IVI subscales.

	Reading IVI		Mobility IVI		Emotional IVI	
	*β* Coefficient	*P* Value	*β* Coefficient	*P* Value	*β* Coefficient	*P* Value
Age	1.414	<**0.001**	1.608	<**0.001**	0.644	**0.017**
**Age-Groups**						
≤75 (Reference)						
>75	21.75	<**0.001**	23.054	<**0.001**	11.475	**0.011**
**Sex**						
Male (Reference)						
Female	−3.550	0.545	2.177	0.716	0.029	0.995
**AMD Stage**						
Early (Reference)						
Intermediate	9.361	0.156	7.709	0.251	6.872	0.299
Late	42.77	<**0.001**	42.438	<**0.001**	27.712	<**0.001**

In univariate linear regression, person measures of all three scales were negatively associated with BCVA, LLVA, MAC-VA and contrast sensitivity in the overall cohort. After controlling for age and AMD stage, multiple regression analysis showed that BCVA and MAC-VA remained significantly associated with all three IVI scores. LLVA was still significantly associated with the reading and mobility scales and contrast sensitivity only with the mobility scale (Table 4). Contrast sensitivity and MAC-VA had the strongest associations with all scales.

**Table 4:** Linear Regression of Visual Function Measures against IVI subscales for the complete cohort.

	Reading IVI		Mobility IVI		Emotional IVI	
	*β* Coefficient	*P* Value	*β* Coefficient	*P* Value	*β* Coefficient	*P* Value
BCVA	−1.030	<0.001*	−1.053	<0.001*	−0.641	<0.001*
LLVA	−0.936	<0.001*	−0.916	<0.001*	−0.558	<0.001
MAC	−1.175	<0.001*	−1.217	<0.001*	−0.781	<0.001*
CS	−1.915	<0.001	−2.094	<0.001*	−1.345	<0.001
IReST^♯^	−0.88	0.393	−0.102	0.303	0.017	0.851
Sensitivity (Mesopic)^♯^	1.094	0.213	0.700	0.412	−0.201	0.807
Sensitivity (Dark-adapted)^♯^	−0.594	0.522	−0.539	0.549	−0.277	0.748

*After controlling for AMD status and age in multiple linear regression, the point estimate remained statistically significant.

BCVA = best-corrected visual acuity, LLVA = low luminance visual acuity, CS = contrast sensitivity, IReST = International Reading Speed Texts.

^♯^Data incomplete.

Analyzing only subjects with early and intermediate AMD, BCVA, LLVA and MAC-VA were associated with the reading scale and BCVA and MAC-VA with the mobility scale. In the adjusted analysis BCVA, LLVA and MAC-VA were still significantly associated with the reading scale and MAC-VA with the mobility scale (Table 5).

**Table 5:** Subgroup Analysis: Linear Regression of Visual Function Measures against IVI subscales for early and intermediate AMD.

	Reading IVI		Mobility IVI		Emotional IVI	
	*β* Coefficient	*P* Value	*β* Coefficient	*P* Value	*β* Coefficient	*P* Value
BCVA	−1.627	0.010*	−1.463	0.033	−0.134	0.806
LLVA	−0.949	0.033*	−0.878	0.069	0.139	0.712
MAC	−1.574	0.011*	−1.651	0.013*	−0.669	0.207
CS	−0.452	0.699	−0.619	0.622	−0.680	0.485
IReST	−0.122	0.399	−0.156	0.301	0.029	0.812
Sensitivity (Mesopic)	1.555	0.210	1.116	0.418	−0.322	0.762
Sensitivity (Dark-adapted)	−0.818	0.532	−0.871	0.548	−0.464	0.677

*After controlling for age in multiple linear regression, the point estimate remained statistically significant.

BCVA = best-corrected visual acuity, LLVA = low luminance visual acuity, CS = contrast sensitivity, IReST = International Reading Speed Texts.

The IReST and macular sensitivity on mesopic and dark-adapted microperimetry showed no association with any of the scales of the IVI.

## Discussion

In our study we found that especially functional tests of central retinal function under low luminance and challenging contrast conditions were associated with VRQoL. LLVA, MAC-VA and contrast sensitivity were significantly reduced in patients with intermediate and late AMD compared to early AMD, while no significant difference was found for BCVA between intermediate and early AMD.

This is in accordance with previous studies, which investigated these tests in early and intermediate AMD, with any visual impairment in early stages of AMD being present on LLVA but not BCVA^33^. Similarly, Feigl and associates reported decreased contrast sensitivity in early AMD compared to healthy controls^34^. In line with previous other studies we found late AMD and older age to be associated with decreased VRQoL on all three subscales, namely the reading, mobility and emotional scales^35,36^. MAC-VA and contrast sensitivity both showed a stronger relationship with the subscales of the IVI in the overall cohort than BCVA. This is of special relevance because poor contrast sensitivity has been shown to be sensitive in discriminating early stages of AMD^34,37^. Furthermore, in previous studies contrast sensitivity was a factor impacting VRQoL of AMD patients: Roh and colleagues^38^ demonstrated that contrast sensitivity was an important factor affecting VRQoL in patients with vision impairment due to bilateral advanced AMD. Bansback *et al*.^39^ found a relationship between contrast sensitivity and health-related quality of life, suggesting that benefits of ocular treatments may be underestimated if contrast sensitivity is not taken into account.

A subgroup analysis, only including early and intermediate AMD subjects, revealed a noticeable association between MAC-VA and VRQoL for both the IVI reading and mobility subscales. The MAC-VA is a relatively recent functional test which simulates lower contrast situations. Shah *et al*.^16^ demonstrated the MAC-VA to be more sensitive in detecting early AMD compared to conventional BCVA and hypothesized that recognition of the high-pass letters is more vulnerable to photoreceptor dysfunction than conventional high luminance and high contrast letters. This is in accordance with our findings as MAC-VA was not only significantly associated with VRQoL in the overall cohort, but also in the subgroup of early and intermediate AMD. As the MAC-VA is a relatively new test, no previous studies have investigated whether it is associated with VRQoL. We could show that MAC-VA as well as LLVA and contrast sensitivity had a stronger effect on VRQoL compared to BCVA. A review by Mones and colleagues revealed that the use of contrast sensitivity as an outcome measure in clinical trials may be a better predictor of activities of daily living, mobility and orientation than BCVA^40^. Also in the Blue Mountains Eye Study, contrast sensitivity was strongly associated with self-reported measures of visual disability^41^.

We did not find significant associations between any of the IVI scales with mesopic or dark-adapted microperimetry mean sensitivity. This is in accordance with the findings of Wu and co-authors^42^. They evaluated subjects with bilateral intermediate AMD using a shorter 10-item Night Vision Questionnaire (NVQ-10)^42^ and assessed the relationship of the NVQ scores with LLVA (also using a standard 2.0.-log neutral density filter), low luminance deficit (LLD), mesopic microperimetric mean sensitivity and central sensitivity. NVQ-10 person measures were significantly associated with LLD, but not with BCVA, LLVA, microperimetric mean sensitivity or central sensitivity. Thompson *et al*., who determined in their study whether Low Luminance Questionnaire scores were associated with objective measures of visual function in early and intermediate AMD, did also not find any association with mesopic microperimetry measures^36^. Interestingly, we did not find reading speed to be correlated with VRQoL.

Strengths of our study include the wide range of functional tests including the relatively new MAC charts for which little data are available to date as well as reading performance and dark-adapted microperimetry. VRQoL was assessed using a validated instrument available in German – the German IVI. We re-evaluated its psychometric performance and transformed responses into an interval-based scale for further statistical testing using modern psychometric methods. Participants were phenotyped based on current gold-standard retinal imaging in combination with a clinical examination. One of the major limitations of our study is the relatively small sample size, especially for the early AMD group. For microperimetry and reading speed measures the sample size was even smaller, as we only performed these test in subjects with early and intermediate AMD. As common with exploratory studies, no adjustment for multiple testing was done which might lead to an over-estimation of statistical power. The IVI assesses overall VRQoL with no particular focus on activities under low luminance and low contrast which might have decreased our ability to detect any associations with functional tests under those conditions. However, general VRQoL is most closely related to daily visual functioning and thus very suitable to assess patient-relevance of visual function tests.

In conclusion, our study showed that performance on BCVA, LLVA, MAC-VA and contrast sensitivity are associated with all aspects of VRQoL in overall AMD. In patients with earlier stages of AMD, BCVA, LLVA and the MAC-VA are associated with VRQoL on the reading scale. In addition, MAC-VA is also correlated with VRQoL on the mobility scale in these patients, which suggests that the MAC-VA might be a useful and patient-relevant measure of visual impairment in AMD, in particular in earlier stages.

## Supplementary information

Supplement 1

Supplement 2

## Data Availability

The datasets generated during and analyzed during the current study are available from the corresponding author on reasonable request.
