# Supplementary material for: Association of Vision-related Quality of Life with Visual Function in Age-Related Macular Degeneration
Source: Sci Rep. 2019 Oct 25;9:15326. doi: 10.1038/s41598-019-51769-7 (PMC6814705; doi:10.1038/s41598-019-51769-7)
Supplement: Supplementary file 1 — Supplement 1 [file 41598_2019_51769_MOESM1_ESM.docx]

**SUPPLEMENTARY INFORMATION**

Association of Vision-related Quality of Life with Visual Function in Age-Related Macular Degeneration

Susanne G. Pondorfer^1^, Jan. H. Terheyden^1^, Manuel Heinemann^1^, Maximilian M.W. Wintergerst^1^, Frank G. Holz^1^, Robert P. Finger^1*^

**^1^**Dept. of Ophthalmology, University of Bonn
Ernst-Abbe-Str. 2, D-53127 Bonn, Germany

*robert.finger@ukbonn.de

**SUPPLEMENT 1**

**Table 6**: Functional tests measures: Descriptive analysis and group comparisons

| **Functional Test**  (Mean [SD]) | **Early AMD** | **iAMD** | **Late AMD** | **P-value* Early vs. iAMD** | **P-value***  **Early vs. late AMD** | **P-value***  **iAMD vs. late** |
| --- | --- | --- | --- | --- | --- | --- |
| BCVA | 84.40 (3.81) | 83.02 (6.78) | 55.84 (14.85) | 0.553 | <0.001 | <0.001 |
| LLVA | 72.00 (4.59) | 65.78 (9.53) | 38.82 (15.73) | 0.023 | <0.001 | <0.001 |
| MAC | 65.70 (3.74) | 60.76 (6.63) | 42.47 (13.65) | 0.041 | <0.001 | <0.001 |
| Contrast Sensitivity | 37.90 (2.81) | 33.33 (3.11) | 24.53 (8.05) | <0.001 | <0.001 | <0.001 |
| IReST | 159.40 (21.37) | 153.84 (29.19) |  | 0.617 |  |  |
| Mesopic Microperimetry | 23.98 (3.04) | 22.95 (3.24) |  | 0.274 |  |  |
| Dark-adapted Microperimetry | 21.28 (4.63) | 20.51 (2.50) |  | 0.141 |  |  |

*P-values based on the Wilcoxon rank sum test, iAMD = intermediate AMD, SD = standard deviation, BCVA = best corrected visual acuity, LLVA = low luminance visual acuity, MAC = Moorfields Vanishing Optotype Charts, IReST = International Reading Speed Text
