# Supplementary material for: Association of Vision-related Quality of Life with Visual Function in Age-Related Macular Degeneration
Source: Sci Rep. 2019 Oct 25;9:15326. doi: 10.1038/s41598-019-51769-7 (PMC6814705; doi:10.1038/s41598-019-51769-7)
Supplement: Supplementary file 2 — Supplement 2 [file 41598_2019_51769_MOESM2_ESM.docx]

**SUPPLEMENTARY INFORMATION**

Association of Vision-related Quality of Life with Visual Function in Age-Related Macular Degeneration

Susanne G. Pondorfer^1^, Jan. H. Terheyden^1^, Manuel Heinemann^1^, Maximilian M.W. Wintergerst^1^, Frank G. Holz^1^, Robert P. Finger^1*^

**^1^**Dept. of Ophthalmology, University of Bonn
Ernst-Abbe-Str. 2, D-53127 Bonn, Germany

*robert.finger@ukbonn.de

**SUPPLEMEMT 2**

During Rasch analysis, the total IVI scale suggested evidence of multidimensionality, with an eigenvalue of 3.38 (although PCA for the first factor explained > 50% of the variance) and three misfitting items, and it was subsequently split into its three component scales: “Reading and Accessing Information” (9 items), “Mobility and Independence” (10 items) and “Emotional well-being” (8 items). For the reading subscale, PSI and PR were 2.28 and 0.84, respectively, indicating that three levels of person strata can be detected. There was minimal evidence of multidimensionality with the PCA for the first factor explaining >60% of the variance and the eigenvalue for the first contrast of 1.86. No DIF was found was for sex and age. The mobility subscale had a PSI of 2.06 and a PR of 0.81. Item 4 displayed misfit, but was retained as its removal did not improve fit statistics. The PCA of the residuals was 58.9%, and the first contrast of the residuals was 1.97 eigenvalue, which is acceptable for the requirements of unidimensionality. No DIF was found for sex but item 19 displayed DIF for age (the younger age group responded differently to this item compared to the older age group >75 years). As we adjusted for age in the regression models we did not remove the item. For the emotional subscale, PSI and PR were 2.02 and 0.80, also indicating that three levels of person strata can be detected. There was no evidence of multidimensionality with the PCA of the residuals of 67.5% and the eigenvalue for the first contrast of 1.85. DIF was found for age, where item 21 had a DIF contrast of 1.47 logits. The item was retained, as it captures important information pertaining to emotional well-being and its removal did not alter the fit statistics relevantly. Targeting was suboptimal for the total IVI scale as well as for the three domains (difference between person and item mean >1.0 for all) indicating that participants had higher ability levels than the mean difficulty of the items.

**Table 7**: Fit parameters of the Complete IVI, Reading IVI, Mobility IVI and Emotional IVI compared with Rasch Model Requirements

| **Parameters** | **Rasch**  **Model** | **IVI_C** | **IVI_R** | **IVI_M** | **IVI_E** |
| --- | --- | --- | --- | --- | --- |
| Misfitting items, n | 0 | **3** (23, 21, 25) | 1 (6) | 1 (4) | 1 (21) |
| PSI | >2.0 | 2.92 | 2.28 | 2.06 | 2.02 |
| PR | >0.8 | 0.90 | 0.84 | 0.81 | 0.80 |
| Difference in person and item mean | <1 | **-1.80** | **-1.58** | **-2.32** | **- 2.45** |
| Variance by the first factor | >50% | 55.6 % | 61.0 % | 58.9% | 67.5 % |
| PCA (eigenvalue for 1st contrast) | <2.0 | **3.38** | 1.86 | 1.97 | 1.85 |
| Differential item functioning  (Item number [DIF contrast]) | <1.0 |  |  |  |  |
| Gender |  | 6 (-1.14) | None | None | None |
| Age group (≤75; ≥76) |  | 19 (1.12)  21 (-1.27)  25 (-1.36) |  | 19 (1.06) | 21 (-1.47) |

IVI_C, complete IVI; IVI_R, Reading and accessing information subscale of the IVI; IVI_M, Mobility and independence subscale of the IVI; IVI_E, Emotional well-being subscale of the IVI. Bold values represent misfit to the Rasch model.
